# Methodological quality of guidelines for management of Lyme neuroborreliosis

**DOI:** 10.1186/s12883-015-0501-3

**Published:** 2015-11-25

**Authors:** R. Dersch, I. Toews, H. Sommer, S. Rauer, J. J. Meerpohl

**Affiliations:** German Cochrane Centre, Medical Center, University of Freiburg, Berliner Allee 29, D-79110, Freiburg, Germany; Department of Neurology, Medical Center, University of Freiburg, Breisacher Str. 64, D-79104, 79106 Freiburg, Germany; Institute of Medical Biometry and Statistics, Medical Center, University of Freiburg, Stefan-Meier-Str. 26, D-79104, Freiburg, Germany

## Abstract

**Background:**

Many aspects of clinical management of Lyme neuroborreliosis are subject to intense debates. Guidelines show considerable variability in their recommendations, leading to divergent treatment regimes. The most pronounced differences in recommendations exist between guidelines from scientific societies and from patient advocacy groups. Assessment of the methodological quality of these contradictory guideline recommendations can be helpful for healthcare professionals.

**Methods:**

Systematic searches were conducted in MEDLINE and databases of four international and national guideline organizations for guidelines on Lyme neuroborreliosis published from 1999–2014. Characteristics (e.g., year of publication, sponsoring organization) and key recommendations were extracted from each guideline. Two independent reviewers assessed the methodological quality of each guideline according to the Appraisal of Guidelines for Research and Evaluation II (AGREE II) tool. AGREE II scores from guidelines developed by scientific societies and from patient advocacy groups were compared across domains.

**Results:**

We identified eight eligible guidelines of which *n* = 6 were developed by scientific societies and *n* = 2 by patient advocacy groups. Agreement on AGREE II scores was good (Cohen’s weighted kappa = 0.87, 95 % CI 0.83–0.92). Three guidelines, all from scientific societies, had an overall quality score of ≥ 50 %. Two of them were recommended for use according to the AGREE II criteria. Across all guidelines, the AGREE II domain with the highest scores was “Clarity of Presentation” (65, SD 19 %); all other domains had scores < 50 % with the domain “Applicability” having the lowest scores (4, SD 4 %). Guidelines developed by scientific societies had statistically significantly higher scores regarding clarity of presentation than guidelines from patient advocacy groups (*p* = 0.0151). No statistically significant differences were found in other domains.

**Conclusions:**

Current guidelines on Lyme neuroborreliosis vary in methodological quality and content. Health care providers and patients need to be aware of this variability in quality when choosing recommendations for their treatment decisions regarding Lyme neuroborreliosis. No statement can be given on quality of content and validity of recommendations, as these issues are not subject to assessment with the AGREE II tool and are prone to individual interpretation of the available evidence by the corresponding guideline panels. To enhance guideline quality, guideline panels should put more emphasis on linking recommendations to the available evidence, transparency in reporting how evidence was searched for and evaluated, and the implementation of recommendations into clinical practice.

**Electronic supplementary material:**

The online version of this article (doi:10.1186/s12883-015-0501-3) contains supplementary material, which is available to authorized users.

## Background

Lyme disease is a tick-borne infectious disease caused by the spirochete bacterium *Borrelia burgdorferi* sensu *lato*. Lyme disease can affect multiple organ systems, common manifestations are dermatologic manifestations (e.g. erythema migrans), Lyme arthritis or Lyme neuroborreliosis [1].

Diagnosis of Lyme disease and Lyme neuroborreliosis is usually based on consensus-derived case definitions [2]. Tiered case definitions exist regarding likelihood of diagnosis depending on diagnostic results [3].

Many aspects of disease management are subject to controversy, sometimes referred to as the ‘Lyme wars’ [4]. Despite the consensus-derived case definitions, controversy further exists on how Lyme disease should be diagnosed and how it should be treated. Some diagnostic tests, like the lymphocyte-transformation test, are discouraged by some guidelines [3, 5, 6], whereas other guidelines recommend the use of this test [7]. What signs and symptoms are suspicious or typical of Lyme disease and which symptoms are rather unspecific is controversial among authors [8, 9]. Therapy of Lyme disease is another subject of disagreements and intense debate, including different opinions regarding choice and dosage of drugs, route of administration and duration of treatment.

These different opinions resulted in different, partially contradicting guideline recommendations for Lyme neuroborreliosis. One example is duration of treatment, for which three guidelines (Infectious Diseases Society of America [IDSA], European Federation of Neurological Societies [EFNS] and the evidence-based practice parameters of the American Academy of Neurology [AAN] [3, 10, 11]) recommend antibiotic treatment with a duration of up to 14–28 days, whereas the guideline of the International Lyme and Associated Diseases Society (ILADS) states that several months of antibiotic therapy are often required [8]. These contradicting recommendations have considerable impact on patient care, as extended antibiotic courses put a high burden on patient adherence, are more expensive, and can even have lethal consequences [12, 13]. The controversy continues with recommendations for antibiotic treatment of residual symptoms after treatment [8], whereas other guidelines discourage repeated antibiotic courses in the absence of ongoing infection, rather recommending symptomatic treatment [3].

Furthermore, guidelines from patient advocacy groups recommend treatment with multiple antibiotic agents and additional adjuvant agents (e.g. hydroxychloroquine) simultaneously [7, 8], whereas other guidelines recommend use of single antibiotic agents [3, 5, 10, 11]. The greatest divergence regarding these contradicting recommendations in different guidelines seems to exist between guidelines developed by scientific societies and guidelines from patient advocacy groups.

These divergent, partly contradicting recommendations lead to uncertainty and doubt in patients and healthcare providers. Evidence from high-quality studies is scarce for treatment of Lyme neuroborreliosis and of limited methodological quality. Hence, contradicting guideline recommendations may exist for conditions with limited evidence, and individual opinions and experiences that are not evidence-based gain increasing influence on guideline recommendations. This, in turn, emphasizes the need for transparent and comprehensible methods for developing guidelines as a sound basis for clinical decisions. To investigate methodological rigour of development of these guidelines with contradicting recommendations, we performed a systematic review focusing on the quality of guidelines for Lyme neuroborreliosis.

## Methods

### Data sources and search strategy

We performed a systematic literature search for guidelines regarding Lyme neuroborreliosis in MEDLINE (via Ovid) and the databases of the National Guideline Clearinghouse (http://www.guideline.gov/), the International Guideline Library of the Guidelines International Network (http://www.g-i-n.net/library/international-guidelines-library), The National Institute for Health and Care Excellence (NICE, http://www.nice.org.uk/guidance/published?type=guidelines) and the Arbeitsgemeinschaft der Wissenschaftlichen Medizinischen Fachgesellschaften (AWMF, http://www.awmf.org/leitlinien/leitlinien-suche.html) from 1999–2014 (Additional file 1). This search was supplemented by records from clinical experts. Language was restricted to English and German due to limited resources.

### Data extraction of guideline characteristics

Two reviewers sequentially extracted relevant information from each eligible guideline: a first reviewer (RD) extracted the data, whereas a second reviewer (IT) checked the first reviewer’s data for completeness and accuracy. Differences in opinion were resolved through discussion. Data was collected on year of publication, country, type of producing organization (scientific society or patient advocacy group), as well as key recommendations for diagnosis and treatment of Lyme neuroborreliosis.

### Assessment of methodological quality of guidelines

Methodological quality was assessed using the Appraisal of Guidelines Research and Evaluation II (AGREE II) instrument [14], which contains 23 items grouped in six domains: 1) scope and purpose; 2) stakeholder involvement; 3) rigour of development; 4) clarity and presentation; 5) applicability and 6) editorial independence. Each domain holds between 2 and 4 items. Two reviewers (RD and IT) independently rated each item on a 7-point scale, with 1 being the lowest and 7 the highest rating. In a consensus meeting among the reviewers, we discussed every item for which the rating differed by more than 1 point (e.g., 1 versus 3) on the original 7-point scale. Reviewers in turn explained the rationale for their rating and had the opportunity to revise it where appropriate. After the consensus meeting, agreement between raters was investigated using Cohen’s weighted kappa and Lin’s concordance correlation coefficient [15].

From the rating, domain scores were calculated as described by AGREE II by the following formula: (obtained score - minimum possible score) / (maximum possible score - minimum possible score) The maximum possible score was: maximum possible score x number of items in domain x number of appraisers. The minimum possible score was: minimum possible score x number of items in domain x number of appraisers. There is no defined threshold for the domain scores of the AGREE II tool to make a distinction between high quality and low quality guidelines, albeit some authors consider domain scores <50 % pragmatically as low quality [16, 17].

According to the AGREE II method, each guideline is rated as either ‘recommended’, ‘recommended with modifications’ or ‘not recommended’ taking into account the appraisal items considered in the assessment process.

If manuals of methodology were accessible for the development process of single guidelines, their content was considered when performing the AGREE II assessment.

As differences in recommendations regarding Lyme neuroborreliosis from different panels were of interest, we compared domain scores between them to investigate whether differences may be paralleled by differences in quality. Additionally, we investigated whether guideline quality was associated with year of publication as an explorative analysis, as guideline quality might increase over time and availability of rating instruments e.g. AGREE II. Statistical comparisons between scores were performed with two-sided t-test. Correlation calculations were performed with Spearman’s rank correlation coefficient. Statistical analyses were conducted with R and Prism 4.0b for Macintosh [18, 19].

As this study does not include any patient data, no ethical approval or consent was needed.

## Results

### Guidelines included

**Fig. 1:**
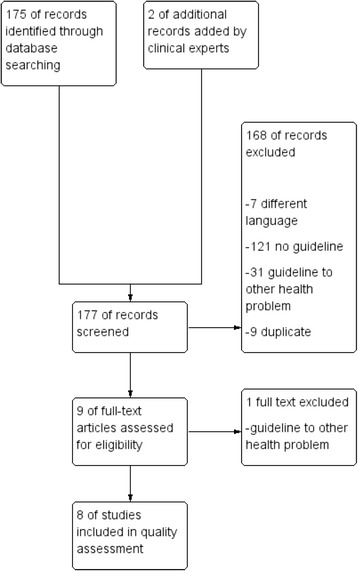
Guideline flow diagram

We identified 177 records; of which 168 were excluded (reasons are listed in Fig. 1). After exclusion of 1 record after full text review, eight eligible guidelines could be included for data extraction and quality assessment. The record excluded in the full text review was an update of another included guideline. As the scope of the updated guideline was diverted from therapy of Lyme neuroborreliosis, the updated guideline no longer fitted our inclusion criteria and was excluded. The original version of the guideline was used for assessment.

Six guidelines were national guidelines. Two guidelines were developed by international organizations [3, 8] (see Table 1).

**Table 1:** Characteristics of included guidelines

Short guideline name	Full guideline name (name of responsible body, if not in title)	Year	Country	Type of organization	Key recommendations for diagnosis	Key recommendations for therapy
AAN	Practice Parameter: Treatment of nervous system Lyme disease (an evidence-based review) (American Academy of Neurology)	2007	USA	Scientific society	None, focus on treatment	Antibiotic regimens for 14 days, either oral or parenteral, doxycycline is the preferred drug for peripheral affections, for more severe manifestations (meningitis, encephalomyelitis) parenteral treatments (ceftriaxone, cefotaxime or penicillin G) are recommended (alternative antibiotics specified)
IDSA	The Clinical Assessment, Treatment, and Prevention of Lyme Disease, Human Granulocytic Anaplasmosis, and Babesiosis: Clinical Practice Guidelines by the Infectious Diseases Society of America	2006	USA	Scientific society	No specific recommendations, focus on treatment	For cranial nerve palsy oral regimen, other neurologic manifestations parenteral regimen for 14-28 days, preferred oral drugs are amoxicillin, doxycycline and cefuroxime, preferred parenteral drug is ceftriaxone (alternative antibiotics specified)
EFNS	EFNS guidelines on the diagnosis and management of European Lyme neuroborreliosis (European Federation of Neurological Societies)	2009	Europe	Scientific society	Investigation of CSF/serum pair for Bb-specific antibodies, intrathecal antibody production and signs of CSF inflammation. Diagnosis according to case definitions (definite/possible).	Early LNB: ceftriaxone IV for 14 days Late LNB: ceftriaxone IV for 21 days Peripheral neuropathy + acrodermatitis chronica atrophicans: doxycycline oral or ceftriaxone IV for 21 days (alternative antibiotics specified)
DGN	S1-Leitlinie Neuroborreliose (German Academy of Neurology)	2012	Germany	Scientific society	Investigation of CSF/serum pair for Bb-specific antibodies, intrathecal antibody production, specific CSF/serum antibody index and signs of CSF inflammation (e.g. pleocytosis). Diagnosis according to case definitions. (definite/probable/possible)	Early LNB: doxycycline oral for 14 days (preferred) Late LNB: ceftriaxone IV for 14-21 days (alternative antibiotics specified)
ILADS	Evidence assessments and guideline recommendations in Lyme disease: the clinical management of known tick bites, erythema migrans rashes and persistent disease (The International Lyme and Associated Diseases Society)	2004	USA	Patient advocacy group	No clear recommendations, emphasis on clinical judgment for diagnosing Lyme disease	No specific recommendations, discussion of a wide range of options, including carbapenems, macrolides, combination of antibiotics and adjuvant treatments (hydroxychloroquine), no clear recommendation for length of treatment but endorsement of longer (>30 days) antibiotic courses
DBG	Diagnostik und Therapie der Lyme-Borreliose – Leitlinien (German Borreliosis Society)	2011	Germany	Patient advocacy group	No clear recommendations, discussion of several diagnostic options	No specific recommendations, discussion of a range of options, including carbapenems, macrolides, metronidazole, combination of antibiotics and adjuvant treatments (hydroxychloroquine). Length of treatment should be at least 28 days, for late LNB 3 months or more.
BIA	The epidemiology, prevention, investigation and treatment of Lyme borreliosis in United Kingdom patients: A position statement by the British Infection Association	2011	UK	Scientific society	Single or paired serum tests for Bb antibodies, intrathecal specific antibody production, specific CSF/serum antibody index, signs of CSF inflammation (e.g. pleocytosis)	Isolated facial nerve palsy or uncomplicated meningitis: doxycycline oral for at least 14 days Complicated meningitis or late LNB: ceftriaxone for 14-28 days (alternative antibiotics specified)
DGPI	Diagnostik und Therapie der Lyme-Borreliose im Kindesalter. Empfehlungen der Deutschen Gesellschaft für Pädiatrische Infektiologie. (Diagnosis and therapy of Lyme borreliosis in children. Recommendations of the German Society of Pediatric Infectiology)	1999	Germany	Scientific society	No clear recommendations, discussion of obligate findings like investigation of CSF/serum pair for Bb-specific antibodies, intrathecal specific antibody production, signs of CSF inflammation (e.g. pleocytosis)	Ceftriaxone, cefotaxime or penicillin G for 14 days

Six of eight included guidelines were developed by scientific societies [3, 5, 6, 10, 11, 20] and two were developed by patient advocacy groups [7, 8]. Constitution of these guideline panels was mostly dominated by neurologists, illustrating the specificity of the underlying topic. No guidelines from other organizations, e.g. public health agencies, were identified. One guideline had a specific focus on recommendations for children with Lyme disease and Lyme neuroborreliosis [20]. Guidelines from patient advocacy groups endorsed extended antibiotic treatments longer than 28 days, whereas guidelines from scientific societies recommended antibiotic treatments for 14–28 days (Table 1).

### Assessment of methodological quality

Discussion on items that were rated >1 point apart by the two raters had to be performed in all domains. Items most often discussed were in domain 1 (‘The overall objective of the guideline is specifically described’, ‘the guideline development group includes individuals from all relevant professional groups’) and in domain 3 (‘the health benefits, side effects, and risks have been considered in formulating the recommendations’).

Inter-rater agreement was high according to Cohen’s weighted kappa (κ = 0.87, 95 % CI 0.83–0.92) and Lin’s concordance correlation coefficient (rho = 0.87, 95 % CI 0.84–0.90). Quality of included guidelines was <50 % in most AGREE II domains; the only domain with a score >50 % was clarity of presentation (mean 65 % of the maximum possible score, SD 19.2 %, Table 2), followed by the domain scope and purpose (mean 45.1 % of the maximum possible score, SD 11.4 %). The domain applicability received the lowest scores across all guidelines (mean 4.6 % of the maximum possible score, SD 4.1 %), followed by editorial independence (mean 15.8 % of the maximum possible score, SD 15.9 %) and rigour of development (mean 18.3 % of the maximum possible score, SD 8.7 %). Three guidelines had a score of ≥50 % in the item overall guideline quality [3, 11].

**Table 2:** AGREEII Domain scores for single guidelines

Short guideline name	Agree domain							
	D1 Scope and purpose	D2 Stakeholders involvement	D3 Rigour of development	D4 Clarity of presentation	D5 Applicability	D 6 Editorial independence	Overall guideline assessment	Recommended^a^
BIA 2010	0.53	0.11	0.09	0.81	0.08	0	0.33	YM
DBG 2010	0.33	0.28	0.10	0.53	0	0.17	0.25	N
DGN 2012	0.28	0.11	0.17	0.64	0.13	0.17	0.33	N
EFNS 2010	0.47	0.17	0.23	0.81	0.04	0.08	0.58	Y
DGPI 1999	0.33	0.14	0.10	0.69	0.06	0	0.25	N
IDSA 2006	0.61	0.5	0.22	0.86	0.02	0.17	0.5	YM
ILADS 2004	0.56	0.36	0.18	0.22	0.04	0.13	0.42	YM
AAN 2007	0.5	0.31	0.37	0.64	0	0.54	0.5	Y
Mean (SD)	0.45 (0.11)	0.25 (0.13)	0.18 (0.08)	0.65 (0.19)	0.05 (0.04)	0.16 (0.16)		n.a.

^a^ *Y* yes, *YM* yes with modifications, *N* = no

Overall two guidelines were rated according to AGREE II rating as ‘recommended by reviewers’, three as ‘recommended with modifications’, and three were ‘not recommended’ (Table 2).

Scores for domain 3 (‘Rigour of development’) correlate with ‚Overall guideline assessment’ (Spearman’s *r* = 0.8537, *p* = 0.0107). Other domain scores did not correlate with ‚Overall guideline assessment’ (not shown). Year of guideline publication did not correlate with any domain score (Table 3).

**Table 3:** Correlation of year of publication and AGREE II domain scores

Domain	Spearman’s r	*p*-value
D1 Scope and purpose	−0.4541	0.2675
D2 Stakeholders involvement	−0.54	0.171
D3 Rigour of development	−0.11	0.793
D4 Clarity of presentation	0.2772	0.5062
D5 Applicability	0.2469	0.5364
D 6 Editorial independence	0.1448	0.7322
Overall quality assessment	0.0248	0.9534

Scope and purpose were often insufficiently described and had to be derived from the title of the guideline in most cases. Specific health questions were described in only one guideline [10], other guidelines addressed generally broad and unspecific health issues.

The domain stakeholder involvement suffered from lack of consideration of views and preferences of the target population.

Systematic methods to search for evidence were only reported in three guidelines [3, 8, 11]. Only one guideline described the applied search strategy in a reproducible way [11]. Criteria for selecting the evidence and the methods of formulating recommendations were described in two guidelines [3, 11], whereas this process remained elusive in the other guidelines.

The link between recommendations and supporting evidence was expressed in most guidelines as a reference to or a narrative review of the respective evidence. A systematic assessment of risk of bias of included studies or a summary of findings table was not presented in any guideline. Only three guidelines rated the risk of bias for the available evidence [3, 10, 11], applying levels of evidence ratings in analogy to the Oxford Centre of Evidence-based Medicine Levels of Evidence [21].

External review was mentioned only in two guidelines [7, 11]. However, it remained unclear who performed the review and how it was performed.

Clarity of presentation was acceptable in most guidelines, although some guidelines presented only vague recommendations with considerable ambiguity in dosages, choice of drugs and on actual length of treatment [7, 8]. Key recommendations were easily identifiable in most guidelines [6, 7, 10, 11, 20], which provided recommendations in a special box or in a separately provided clinical pathway [5]. Other guidelines presented recommendations embedded in the continuous text, which were more difficult to identify [8].

Applicability was insufficiently addressed in almost every guideline. Potential barriers or resource implications, like availability of specialized laboratories for valid serologic testing, were addressed in only three guidelines [3, 5, 6]. Monitoring and auditing criteria were not mentioned in any guideline.

Conflicts of interest were not disclosed in three guidelines [6, 8, 20]. The other guidelines disclosed conflicts of interest, but it remained unclear how potential influences of these conflicts on recommendations were operated.

Guidelines developed by scientific societies scored different results than guidelines developed by patient advocacy groups. Results are shown in Table 4. Guidelines developed by scientific societies had statistically significantly higher scores for clarity of presentation (*p* = 0.0151), differences in other domains were not statistically significant. Overall guideline assessment was not significantly different for guidelines from scientific societies and from patient advocacy groups (*p* = 0.4534, Table 4).

**Table 4:** Comparison of AGREEII domain scores for guidelines developed by scientific societies and by patient advocacy groups

Domain	Scientific society		Advocacy group		*p*- value
	Mean	SD (range)	Mean	SD (range)	
D1 Scope and purpose	0.478	0.109	0.445	0.115	0.7761
D2 Stakeholders involvement	0.24	0.0149	0.32	0.04	0.5732
D3 Rigour of development	0.216	0.092	0.14	0.04	0.2763
D4 Clarity of presentation	0.752	0.093	0.375	0.155	0.0151*
D5 Applicability	0.054	0.046	0.02	0.02	0.4358
D 6 Editorial independence	0.192	0.1852	0.15	0.02	0.7976
Overall guideline assessment	0.42	0.12	0.33	0.83	0.4534

*p < 0.05

## Discussion

Methodological quality of existing guidelines for treatment of Lyme neuroborreliosis is limited and shows considerable variability across individual guidelines identified. Quality assessments of many domains were unsatisfactory according to the AGREE II tool. Quality scores partly differed between guidelines developed by scientific societies and guidelines developed by patient advocacy groups, with statistically significant differences in clarity of presentation. Interestingly enough, year of publication did not correlate with any of the quality score, albeit this could be to the low sample size. Due to language limitations, we may have missed guidelines which could not be assessed in this review.

Discussion on items that were rated >1 point apart by the two raters had to be performed in all domains, mostly because information on single items was scattered through individual guidelines and was difficult to gather. Such disagreements could easily be cleared in the consensus discussion.

Quality assessment of guidelines with the AGREE II tool covers issues of methodological rigour of guideline development, applicability and transparency. No statement can be given on quality of content and validity of recommendations drawn from the available body of evidence, as these issues are not subject to assessment with the AGREE II tool and are prone to individual interpretation of the available evidence by the corresponding guideline panels. However, as these issues cannot be assessed directly, the process of selecting evidence and linking of recommendations to supporting evidence in an individual guideline should be transparent and comprehensible, which was insufficient in most included guidelines. The importance of methodological rigour of development for guideline development is illustrated by our finding that the domain score for ‘rigour of development’ correlated statistically significantly with the overall guideline assessment.

Whereas clarity of presentation was acceptable in some guidelines, more emphasis on rigour of development and especially systematic search methods, criteria for selecting evidence and linking of recommendations to supporting evidence as well as addressing applicability issues could lead to improved quality and better usability of guidelines for management of Lyme disease. The credibility of a guideline is diminished when it is not clear whether systematic search methods were used to gather available evidence or how recommendations are linked to supporting evidence.

Linkage of recommendations to the available evidence could be improved by implementing summary of findings tables according to the GRADE approach [22]. Readers could then identify key recommendations more easily and would be provided with additional information on the strengths and weaknesses of the body of evidence.

Applicability of guidelines could be improved by providing advice or tools on how the recommendations can be put into practice, e.g. short versions, clinical pathways or clear accentuation of key recommendations. Potential resource implications could be applied, e.g. the need for specialized laboratories on serologic testing.

Monitoring and auditing criteria were not mentioned in any guideline, although it may be difficult to determine such criteria for treatment of Lyme neuroborreliosis. Included guidelines did not intend to provide monitoring criteria but rather stated that the intention was to provide support and guidance for clinicians and patients in treatment decision.

In the light of contradicting recommendations in a field with limited evidence, it seems necessary to provide guidelines which are transparently developed and provide evidence based recommendations for clinicians. Individual guidelines with highest overall scores on guideline quality, which were also the two guidelines recommended according to AGREE II ratings were the EFNS and the AAN guidelines.

These two guidelines that were ‘recommended’ according to AGREE II had especially high scores in the domains ‘rigour of development’ and ‘clarity of presentation’ compared to the guidelines that were not recommended. All other guidelines, including the current guideline from the DGN, were not rated as ‘recommended’.

Both guidelines stem from scientific societies. None of the guidelines from patient advocacy groups were rated as ‘recommended’. Guidelines from patient advocacy groups endorsed extended antibiotic treatments longer than 28 days, whereas guidelines from scientific societies recommended antibiotic treatments for 14−28 days. Differences in these recommendations might partly be explained by differences in methodological quality of guidelines.

## Conclusions

Clinicians and patients faced with treatment decisions on Lyme neuroborreliosis can use the provided quality assessment of the available guidelines to choose individual guidelines showing high methodological quality according to the AGREE II tool.

## Additional file

Additional file 1:
**Search strategy MEDLINE (OVID).** (DOCX 46 kb)
