# Supplementary material for: Methodological quality of guidelines for management of Lyme neuroborreliosis
Source: BMC Neurol. 2015 Nov 25;15:242. doi: 10.1186/s12883-015-0501-3 (PMC4660677; doi:10.1186/s12883-015-0501-3)
Supplement: Additional file 1: — Search strategy MEDLINE (OVID). (DOCX 46 kb) [file 12883_2015_501_MOESM1_ESM.docx]

Appendix 1: search strategy MEDLINE (OVID)

1. exp Lyme Disease/

2. lyme*.mp.

3. exp Borrelia burgdorferi Group/

4. borrel*.mp.

5. 1 or 2 or 3 or 4

6. exp practice guideline/

7. Health Planning Guidelines/

8. guideline*.ti.

9. (practice adj3 parameter*).ti,ab.

10. clinical protocols/

11. guidance.ti,ab.

12. care pathway*.ti,ab.

13. critical pathway/

14. (clinical adj3 pathway*).ti,ab.

15. algorithms/

16. consensus development conference.pt.

17. consensus development conference nih.pt.

18. 6 or 7 or 8 or 9 or 10 or 11 or 12 or 13 or 14 or 15 or 16 or 17

19. 5 and 18
